# Peripheral proteinopathy in neurodegenerative diseases

**DOI:** 10.1186/s40035-024-00461-6

**Published:** 2025-01-16

**Authors:** Bin Xu, Xia Lei, Ying Yang, Jiayi Yu, Jun Chen, Zhi Xu, Keqiang Ye, Jing Zhang

**Affiliations:** 1 Department of Pathology, School of Medicine, The First Affiliated Hospital, Zhejiang University, Hangzhou, 310003 China; 2 School of Brain Science and Brain Medicine, Zhejiang University, Hangzhou, 310002 China; 3 Department of Pathology and Laboratory Medicine, Emory University School of Medicine, Atlanta, GA 30322 USA; 4 Faculty of Life and Health Sciences, Shenzhen University of Advanced Technology (SUAT), Shenzhen, 518055 China; 5 National Human Brain Bank for Health and Disease, Zhejiang University, Hangzhou, 310012 China

**Keywords:** Neurodegenerative diseases, Peripheral proteinopathies, Amyloid β, Tau, α-Synuclein, TDP-43

## Abstract

Proteinopathies in neurology typically refer to pathological changes in proteins associated with neurological diseases, such as the aggregation of amyloid β and Tau in Alzheimer’s disease, α-synuclein in Parkinson’s disease and multiple system atrophy, and TAR DNA-binding protein 43 in amyotrophic lateral sclerosis and frontotemporal dementia. Interestingly, these proteins are also commonly found in peripheral tissues, raising important questions about their roles in neurological disorders. Multiple studies have shown that peripherally derived pathological proteins not only travel to the brain through various routes, aggravating brain pathology, but also contribute significantly to peripheral dysfunction, highlighting their crucial impact on neurological diseases. Investigating how these peripherally derived proteins influence the progression of neurological disorders could open new horizons for achieving early diagnosis and treatment. This review summarizes the distribution, transportation pathways, and pathogenic mechanisms of several neurodegenerative disease-related pathological proteins in the periphery, proposing that targeting these peripheral pathological proteins could be a promising strategy for preventing and managing neurological diseases.

## Introduction

Neurodegenerative diseases encompass a range of conditions impacting the central nervous system (CNS), including Alzheimer’s disease (AD), Parkinson’s disease (PD), multiple system atrophy (MSA), amyotrophic lateral sclerosis (ALS), frontotemporal dementia (FTD), and several other diseases. These diseases typically result from the abnormal aggregation of proteins in the CNS, such as amyloid-β (Aβ) plaques and tubulin-associated unit (Tau) for AD, α-synuclein (α-syn) in neurons for PD, α-syn in oligodendrocytes for MSA, TAR DNA-binding protein 43 (TDP-43) for ALS and a type of FTD, leading to gradual deterioration of neural structure and function [1–4]. With the increasing aging population globally, the incidence of these diseases is escalating annually, presenting serious challenges to global public health [5, 6]. Current therapies primarily focus on managing symptoms to improve the quality of life for patients [7, 8]. However, achieving a fundamental cure remains challenging. Therefore, it is urgent and imperative to explore the deeper mechanisms underlying neurodegenerative diseases and to develop corresponding treatments targeting these mechanisms.

While traditional research has primarily focused on the pathological proteins in the CNS [9], increasing evidence from human proteomics research indicates that these proteins are also expressed in various peripheral organs [10–12]. Recently, increasing studies have demonstrated that peripheral tissues, such as the gastrointestinal tract, express pathological proteins at elevated levels in patients with neurodegenerative disease, highlighting their potential role in driving disease progression [13–15]. The peripheral pathological proteins have been confirmed to not only be transported to the brain, inducing CNS pathology, but also to play a role in regulating homeostasis within the peripheral system.

This review summarizes the peripheral distribution and physiological functions of Aβ, Tau, α-syn, and TDP-43, the potential mechanisms of their transportation into the brain, and their roles in the pathogenesis of neurodegenerative diseases. In addition, we discuss therapeutic approaches targeting peripheral pathological proteins in neurodegenerative diseases. Our review contributes to comprehending the relationship between peripheral proteinopathies and neurodegenerative diseases, providing novel insights and strategies for their prevention and treatment.

## Origins and functions of peripheral key proteins

### Distribution and function of peripheral Aβ

**Fig. 1:**
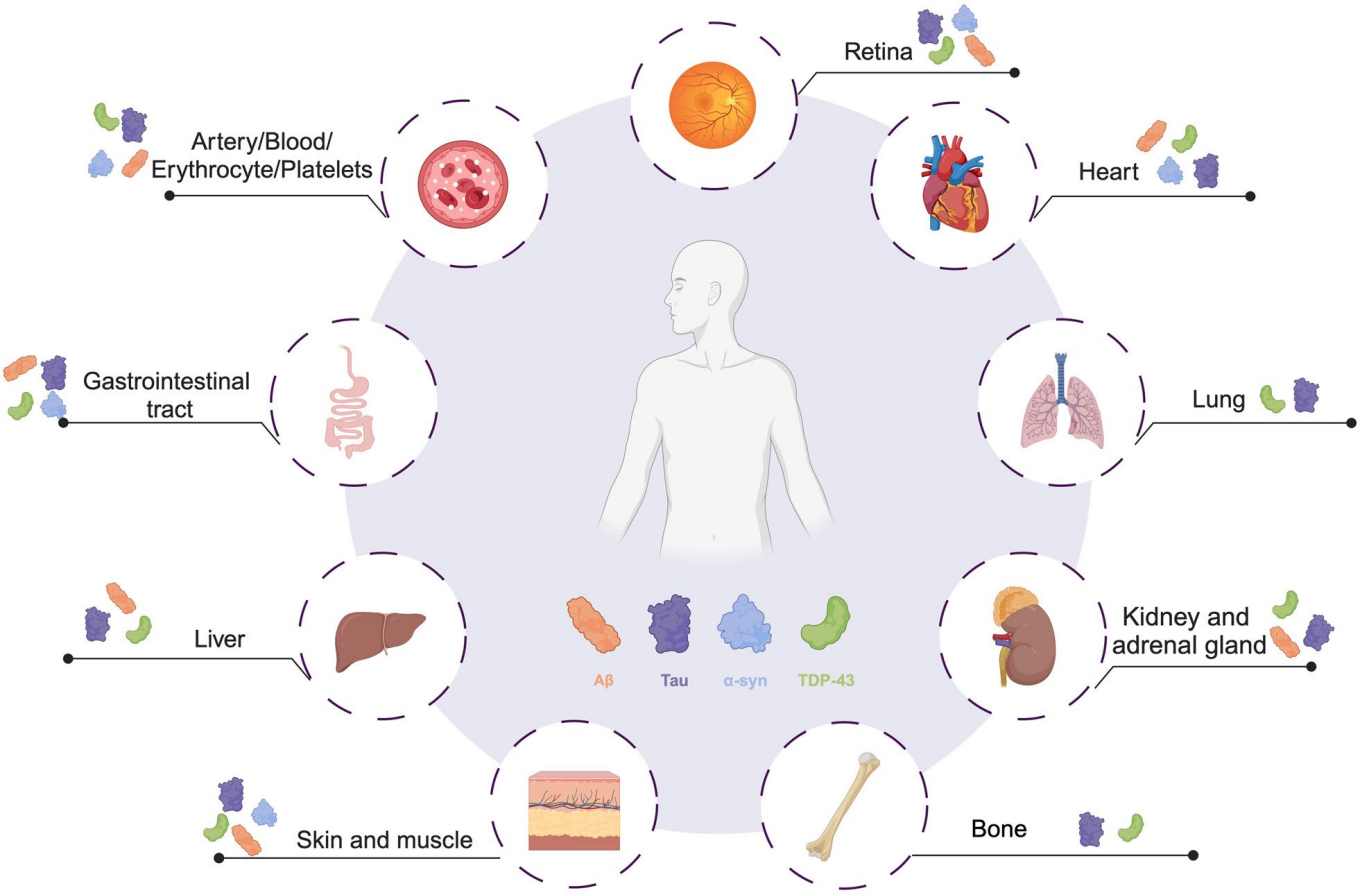
The distribution of neurodegenerative disease-related pathological proteins in peripheral tissues. The characteristic pathological proteins of neurodegenerative diseases, including Aβ, Tau, α-syn and TDP-43, are not only expressed in the CNS but are also widely present in peripheral tissues such as blood, gastrointestinal tract, liver, kidney, lung, heart, skin, muscles, bone, and peripheral nerves. The levels of these pathological proteins in the periphery are regulated by the peripheral environment including bacteria and inflammation. These proteins perform various physiological and pathological functions in peripheral tissues

Aβ was initially characterized by Dr. Glenner and Dr. Wong from the brain blood vessels of AD patients [16], and several subsequent studies have affirmed that Aβ is one of the key pathological features of AD [17, 18]. While Aβ is primarily produced in the brain, studies have found that a wide range of peripheral tissues such as the liver, kidney, spleen, intestine, salivary gland, adrenal gland, heart, pancreas, thyroid gland, endothelial lymph nodes, retina, skeletal muscle, skin and various blood cells also extensively express the amyloid precursor protein (*APP*) gene, with corresponding production of peripheral APP and related peptides (Fig. 1, Table 1) [19–21]. In the skeletal muscle, although the amount of Aβ produced by skeletal muscle cells is relatively small, nearly one-third of the total body weight of skeletal muscle still expresses high levels of Aβ in peripheral tissues [22, 23]. In human peripheral blood, cells can internalize APP and produce peripheral Aβ, with platelets being the major source, accounting for over 90% of Aβ [24, 25]. Differential expression of Aβ in the skin has been observed in AD patients, and Aβ aggregates may form in the skin or subcutaneous tissue [26]. The liver and kidney are the main organs responsible for Aβ metabolism and clearance in the body, while their hepatocytes and renal tubular epithelial cells can also produce APP and break it down into Aβ [27, 28]. The intestine is another important organ with high expression of peripheral Aβ. Studies have reported elevated intestinal Aβ aggregates in AD patients, possibly contributing to the pathological progression of AD [29]. It should be noted that while multiple peripheral tissues can express the *APP* gene and produce Aβ, the predominant form of Aβ generated by peripheral tissue proteolysis is Aβ_40_, which is different from the more neurotoxic Aβ_42_ subtype synthesized primarily by CNS cells [30–33], with much less Aβ aggregation in peripheral tissues at least in physiological conditions. The differential extent of Aβ aggregation between the peripheral organs and brain may also be attributed to the different isoforms of APP in the periphery versus the CNS [33], ranging in size from 695 to 770 amino acids. The most abundant form in the brain (APP_695_) is produced mainly by neurons and differs from longer forms of APP expressed by peripheral cells and platelets (APP_751_ and APP_770_ isoforms) in that the CNS form lacks a Kunitz-type protease inhibitor sequence in its ectodomain [34, 35].

**Table 1:** Summary of recent studies on protein properties in central and peripheral systems

Proteins		Gene	Isoforms	Distribution and aggregation	References
APP	APP_695_	*APP*	Aβ_42_	Brain (extracellular matrix)^*O,F,D*^	[17, 18, 41]
	APP_751_ & APP_770_		Aβ_40_	Skin^*D*^, subcutaneous tissue^*D*^, liver (hepatocytes)^*D*^, intestine^*D*^, blood (platelet) and blood vessels^*D*^, retina^*D*^, kidney (renal tubular epithelial cells)^*D*^, muscle (skeletal muscle cells)^*D*^, and heart^*D*^	[16, 36–39]
Tau		*MAPT*	Small Tau	Brain (neurons)^*NFT*^	[74, 75]
			Big Tau	Peripheral nervous system (peripheral neurons)^*D*^, blood (monocytes and lymphocytes), lungs (endothelial cells)^*D*^, heart^*D*^, liver^*D*^, skin^*D*^, kidney^*D*^, pancreas (pancreatic beta cells)^*D*^, intestine (enteroendocrine cells)^*D*^, gallbladder^*D*^, submandibular gland^*D*^, retina^*D*^, bone^*D*^, and muscle^*D*^	[63–71, 78, 79]
α-Syn		*SNCA*	Non-α-helix structure	Brain (neurons and oligodendrocytes)^*O,F,D*^	[84, 85]
			α-Helix structure	Intestine (enteric neurons), peripheral nervous, heart, blood (erythrocytes), skin, retina^*D*^, and submandibular gland	[86–93, 97, 98, 100, 101]
TDP-43		*TARDBP*	TDP-43	Brain^*O,F,D*^	[103–113]
				Throughout the body (cell nucleus) skeletal muscle^*D*^, cardiac muscle^*D*^, paraspinal muscle^*D*^, diaphragm^*D*^, appendicular muscle^*D*^, intramuscular nerve bundle^*D*^	

*D: Deposits; O: Oligomers; F: Fibrils; NFT: Neurofibrillary tangles*

Despite the difference in isoforms, Joachim et al. have reported that Aβ deposits are detected in non-neural tissues, including skin, subcutaneous tissue, intestine and blood vessels, of AD patients and elderly normal subjects [36]. Similarly, aggregated Aβ has been found in the impaired myocardial tissue and retina of AD patients [37, 38]. Additionally, amyloid-like deposits accumulate during natural aging in the body wall muscle of *C. elegans*, in human primary myotubes, and in mouse skeletal muscle [39]. The support for Aβ aggregation can also be found in the reports that β- and γ-secretases, two enzymes essential for the generation of the toxic Aβ species [40, 41], are present in peripheral organs. Specifically, β-secretase (BACE1) mRNA and protein are found in many cell types including pancreatic β-cells, adipocytes, hepatocytes, and vascular cells [42]. γ-Secretase is a multiprotein complex consisting of presenilin, nicastrin, anterior-pharynx defective-1, and presenilin enhancer-2 [43]. γ-Secretase is clearly detected in the spleen, kidneys, lungs, pancreas, and liver, although, like BACE1, its expression level is much higher in the brain and the spinal cord [44].

While Aβ and its aggregates are found in various peripheral tissues and cells of the human body, the physiological functions of peripheral Aβ have not been fully elucidated. Research has discovered the antimicrobial activity of Aβ by inhibiting the growth and proliferation of bacteria in gut [45, 46]. Conversely, bacteria can also influence the expression and aggregation of Aβ [47]. Transplanting fecal microbiota from APP/PS1 mice into wild-type mice significantly increases intestinal Aβ expression, exacerbating neuroinflammation and Aβ plaques in the CNS. In contrast, transplantation of fecal microbiota from healthy wild-type mice ameliorates AD pathological changes in ADLP^APT^ transgenic mice [48]. Numerous studies have reported that the dysbiosis of gut microbiota in AD patients leads to abnormal elevation of Aβ expression in the intestine [49–51]. Additionally, Aβ has potential anti-cancer effects [52] although the underlying mechanisms are not fully understood. Studies have shown that Aβ can inhibit the growth of human glioblastoma and adenocarcinoma tumors by regulating the cell cycle, suppressing tumor angiogenesis, inducing apoptosis of tumor cells and reducing the blood supply needed for tumor growth [53].

The immunomodulatory effects of peripheral Aβ are controversial and not yet fully confirmed. Some studies have shown that Aβ can influence the activity and function of macrophages in APP/PS1 mice, regulating the release of inflammatory factors and affecting the body’s inflammatory response [54, 55]. Li et al. found that Aβ exerts a biphasic effect at different stages of AD, suppressing peripheral inflammatory macrophages in the early stage of the disease while promoting them in the late stage [54, 55]. Aβ also affects the function of T cells, B cells, and other immune cells in human immune system, including cell proliferation, differentiation, and activation, thereby influencing the immune response [56, 57]. However, there is still significant debate on the exact mechanisms of immune regulation by Aβ and the impact of peripheral Aβ on brain immunity and AD pathogenesis (see below for more detailed discussion).

### Distribution and function of peripheral Tau

Tau, first discovered by Dr. Kirschner’s laboratory in 1975 [58], is a microtubule-associated protein primarily expressed in neurons, where it plays a role in microtubule stabilization and axonal transport [59]. The CNS Tau is encoded by 6-kb mRNA and exists as six isoforms with molecular weights ranging from 48 to 67 kDa. In contrast, peripheral Tau, often referred to as ‘big Tau’, is a larger high-molecular-weight isoform (approximately 110 kDa) produced from an alternative splicing of the Tau mRNA transcript, resulting in an 8-kb mRNA variant. All of these isoforms are encoded by the same gene [60]. In the peripheral ganglia of neonatal animals, both 6-kb and 8-kb tau mRNAs are present, whereas in adult animals, only the 8-kb mRNA is detected peripherally [61]. This indicates that Tau mRNA undergoes different splicing in the central and peripheral nervous systems of vertebrates, with the big Tau being specifically expressed in the peripheral nervous system [62]. Tau is found in many peripheral tissues such as the peripheral nervous system, lungs, heart, intestines, gallbladders, submandibular glands, kidneys, bone, muscles, skin, and retina (Fig. 1, Table 1) [63–69]. Peripheral nerves, including those in the heart and intestines, are the primary sources of peripheral Tau [70]. In addition to peripheral neurons, cardiomyocytes in the heart, epithelial cells in the lungs, and enteroendocrine cells in the intestines can also produce Tau [12, 64, 71].

Peripheral big Tau undergoes post-translational modifications, such as phosphorylation and acetylation [72]. Dysregulation of these processes may contribute to the formation of hyperphosphorylated and misfolded Tau, which can then enter the circulation and potentially the CNS [73]. To this end, abnormal phosphorylation and aggregation of CNS Tau is a critical pathological mechanism in AD [74, 75]. However, it is more difficult for peripheral big Tau to form aggregates, due to its complex structural characteristics compared to the CNS Tau [76, 77]. The precise impact of peripheral big Tau on CNS Tau in neurodegeneration remains to be fully understood. Additionally, the influence of big Tau species on the detection of phosphorylated peripheral Tau as a biomarker requires further investigation. For example, Tau hyperphosphorylation at Ser396 has been detected in the hearts of patients with heart failure and AD, which may lead to formation of toxic oligomers [78, 79].

In peripheral tissues, Tau may have functions beyond microtubule stabilization. For instance, the expression of Tau increases in pulmonary endothelial cells during bacterial pneumonia and sepsis [80, 81]. Although the specific pathological mechanisms are unclear, a series of studies have demonstrated that abnormalities in Tau can lead to skeletal muscle dysfunction and result in muscle weakness [82]. Targeting Tau through immunotherapy improves myocardial function, indicating that the pathological aggregation of Tau disrupts cardiac function [78, 79].

### Distribution and function of peripheral α-syn

Traditionally, α-syn is believed to be a relatively small, soluble protein primarily expressed in the CNS, where it is involved in synaptic vesicle trafficking and neurotransmitter release [83]. The aggregation of α-syn-containing Lewy bodies in neurons or oligodendrocytes is a major pathological feature of PD or MSA [84, 85]. Though first discovered in the brain by Dr. Maroteaux, the protein is widely distributed in various tissues throughout the body (Fig. 1, Table 1) [86–88]. Studies have shown that α-syn is abundantly expressed in the intestine, especially in enteric neurons [89], and regulates gastrointestinal motility and secretion [90]. Its expression has also been detected in the heart, indicating its potential involvement in cardiovascular regulation [91]. Although *SNCA* mRNA is not expressed in the liver, α-syn protein accumulates there and serves as a major pathway for α-syn metabolism [92]. Additionally, over 99% of α-syn in the blood is carried by erythrocytes, making them an important source of peripheral α-syn [93]. It should be noted that due to structural differences between α-syn in peripheral red blood cells and the CNS, erythrocytic α-syn, which predominantly exists as stable, helically folded tetramers, is less prone to aggregation compared to brain α-syn [94, 95].

The physiological mechanisms of α-syn in the periphery have not been fully elucidated. α-Syn has physiological effects on the skin, regulating the proliferation and differentiation of skin cells and maintaining the skin barrier and skin nerve [96, 97]. Additionally, α-syn participates in immune regulation in the skin, modulating skin inflammation and immune responses [98]. The widespread expression of α-syn in the periphery is important evidence for the peripheral origin hypothesis of PD. Several factors such as inflammation, oxidative stress, and aging can promote the misfolding and aggregation of α-syn in peripheral tissues, which may then spread to the CNS via various routes [99]. Intestinal α-syn also causes gastrointestinal dysfunction in patients with PD [100]. Yang et al. found that α-syn can spontaneously aggregate in the intestine [101]. Challis et al. demonstrated that injection of α-syn fibrils into the gut can cause gastrointestinal impairment [102]. Further investigations are required to clarify whether peripheral aggregation of α-syn in other organs causes tissue damage or can be transported to the CNS.

### Distribution and function of peripheral TDP-43

TDP-43, encoded by the *TARDBP* gene [103], was initially discovered in 1995 as a suppressor of HIV-1 gene expression. It is a highly conserved RNA/DNA-binding protein expressed throughout the body (Fig. 1, Table 1). In physiological conditions, TDP-43 is predominantly localized in the nucleus. However, it can shuttle between the nucleus and cytoplasm through active and passive transport, exerting physiological functions in the cytoplasm, including the regulation of mRNA transport, RNA metabolism, microRNA maturation, and stress granule formation [104]. TDP-43 is also found in mitochondria, where it interacts with the mitochondrial genome and plays a crucial role in respiratory chain pathways [105]. TDP-43 is essential for early embryogenesis as knockout of *TARDBP* leads to embryonic lethality in mice [106]. The mislocalization and aggregation of TDP-43 are considered key pathologies underlying neurodegenerative diseases including ALS and FTD [107]. Although no studies have yet reported whether there are differences between the central and peripheral isoforms of TDP-43, an increasing number of studies have begun to focus on the abnormal distribution and expression of TDP-43 in peripheral tissues (Table 1). TDP-43 aggregates are found in skeletal muscles of patients with neuromuscular diseases such as sporadic inclusion body myositis, familial ALS, and polymyositis [108, 109]. Recent autopsy studies have identified phosphorylated TDP-43 (pTDP-43, pSer409/410) aggregates in the skeletal and cardiac muscles as potential biomarkers for both ALS and non-ALS, such as sporadic inclusion body myositis, myofibrillar myopathies, hereditary motor and sensory neuropathy, etc [110, 111]. Further investigation indicates that pTDP-43 pathology is more commonly observed in axial muscles, i.e., the paraspinal muscles and diaphragm, and less frequently in appendicular muscles [112]. Moreover, pTDP-43 aggregation is also present in the intramuscular nerve bundle axons of ALS patients, suggesting that pTDP-43 in the intramuscular nerve bundles may be a peripheral pathological feature of ALS [113]. Despite these exciting discoveries, our understanding of the peripheral TDP-43 in normal and diseased conditions, such as ALS and FTD, is rather limited.

## Transportation of peripheral key proteins to the brain

**Fig. 2:**
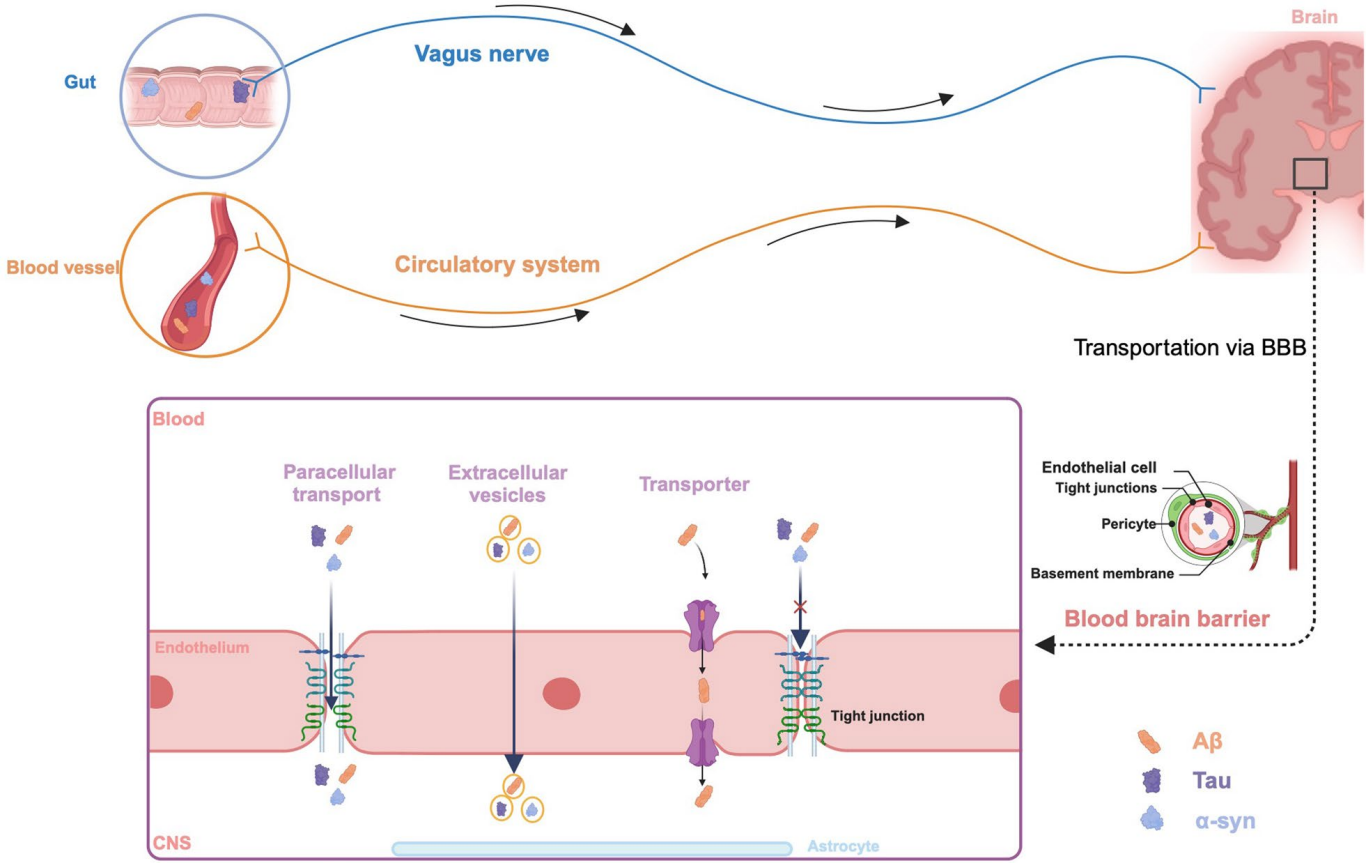
Transportation of peripheral pathological proteins into the brain. Peripheral pathological proteins can enter the brain through various pathways. Aβ, Tau, and α-syn enriched in the gastrointestinal tract can be transported via the vagus nerve. On the other hand, these pathological proteins can flow through the bloodstream from the periphery into the CNS. When the BBB remains intact, these proteins can cross into the CNS through specific transporters, such as AGER, which selectively transports Aβ. Additionally, EVs carrying these pathological proteins can be endocytosed by brain microvascular endothelial cells, allowing them to cross the BBB. When the BBB is compromised, these pathological proteins, whether in free form or encapsulated within EVs, can infiltrate the brain through the intercellular spaces

Before considering the influence of peripheral proteinopathies on the CNS, it is essential to understand the routes by which they are transported into the brain. All peripheral proteins discussed above can exhibit prion-like biophysical and biochemical properties [114, 115]. Unlike conventional viruses, prions lack genetic material (such as DNA or RNA) and propagate by inducing misfolding of normal proteins into an abnormal conformation, a process known as "seeding" [116, 117]. Many misfolded gut-derived proteins, including Aβ, Tau, and α-syn, have been shown to transmit to the brain via the vagal nerve, leading to neuronal death (Fig. 2) [118–122], through a prion-like mechanism. Additionally, these peripherally derived proteins can enter the brain directly via transporters on the blood–brain barrier (BBB) or through extracellular vesicles (EVs) secreted by peripheral tissue cells, especially when the BBB is compromised (Fig. 2) [123, 124] (see below for more detailed discussion). Much less is known about TDP-43 transmission. Finally, through transportation via the gut-brain axis, Aβ, Tau, α-syn, and TDP-43 derived from dietary sources or the gut microbiota may play a role in the pathogenesis of neurodegenerative diseases.

### Vagus nerve

Peripheral Aβ, Tau, and α-syn, especially those derived from the intestine, can enter the brain through the vagus nerve, which is an important pathway in the pathogenesis of neurodegenerative diseases [121, 122, 125]. The vagus nerve, one of the longest peripheral nerves, originates from the brainstem and extends to the abdomen and pelvis, controlling the function of multiple visceral organs [126]. It can serve as a conduit for the spread of misfolded proteins from the periphery to the CNS. Several studies have found that intestinal injection of Aβ, Tau, and α-syn can promote the transportation of pathological proteins from the peripheral system to the CNS via the vagus nerve. Notably, severing the vagus nerve significantly inhibits the entry of these injected proteins into the brain [127–129]. We have also shown that gut Aβ can lead to neuronal damage, which can be alleviated by vagotomy, although the process seems to be age-dependent [130]. In addition to exogenously injecting these pathological proteins, Xiang et al. using newly developed gut-inducible SYN103^+/-^ and TAU368^+/-^ transgenic mouse models, further demonstrated that endogenous gut-derived α-syn and Tau can spontaneously aggregate and propagate to the brain via the vagus nerve [131]. However, it should be noted that some have suggested that vagal nerve transmission may not be significant in non-human primates [132], which could be attributed to factors such as the animal model used, the type of inoculum, the time after injection, and species differences.

### BBB and transporter

Besides the vagus nerve, soluble pathological proteins in the circulatory system can directly interact with the transporter on the recipient cells. BBB plays a critical role in regulating the exchange of molecules between the peripheral circulation and the CNS [133]. Under normal conditions, the intact BBB effectively prevents the transport of large peripheral molecules into the brain. However, in pathological conditions such as aging, trauma, and inflammation, peripheral pathological proteins including Aβ, Tau, and α-syn can enter the brain through enhanced active transcytosis, including receptor-mediated and absorptive transcytosis, as well as passive diffusion through damaged BBB [134]. The increased transcytosis may be attributed to the upregulation of receptors, as recent research revealed that elevated expression of *AGER*, which encodes the receptor for advanced glycation end-products, in endothelial cells, promotes the transport of peripheral Aβ to the brain [135]. Specific transporters for other peripheral pathological proteins into the brain have not yet been identified. Further research is needed to uncover more transporters and their roles in the pathological progression of neurodegenerative diseases.

### EVs

EVs are membrane-bound small vesicles produced by cells, playing a crucial role in intercellular communication and signaling by containing substances such as proteins, nucleic acids, and lipids [136]. In recent years, EVs have received increasing attention due to their ability to traverse the intact BBB. For example, erythrocyte-derived EVs carrying α-syn enter brain through the BBB [137–139]. By radioactively labeling red blood cell-derived EVs (RBC-EVs) with Na^125^I and DiI labeling, Matsumoto et al. found that these EVs can enter the brain even when the BBB is intact and are primarily phagocytosed by microglia [139]. Subsequently, in vivo imaging was conducted to track DiR-labeled RBC-EVs injected via the tail vein and radioactive ^125^I-labeled RBC-EVs injected into the gut, further confirming that the peripheral EVs can transfer to the brain [101, 140]. Immunostaining for DiI revealed that these RBC-EVs were captured by neurons and astrocytic end-feet [101, 140]. These studies also found that BBB disruption by lipopolysaccharide accelerates the entry of peripheral RBC-EVs into the brain [101, 139, 140]. These findings suggest that EVs may be one of the important pathways for peripheral proteins to cross the BBB and enter brain tissue, which is significant for understanding the pathogenesis of neurodegenerative diseases and identifying new therapeutic targets. However, the entry of peripheral EVs carrying Aβ, Tau and TDP-43 into the brain has not yet been confirmed. Further studies are required to validate the potential of other pathological proteins to enter the brain via EVs, as well as their exact roles and potential applications in neurodegenerative diseases.

## Neuropathology

### AD

Several studies have reported that peripheral Aβ and Tau play significant pathogenic roles in AD, with particular emphasis on Aβ produced in the gut, which is regulated by the gut microbiota. Gut-derived Aβ can enter the brain through various pathways and is believed to be an important pathogenic factor in the progression of AD.

**Fig. 3:**
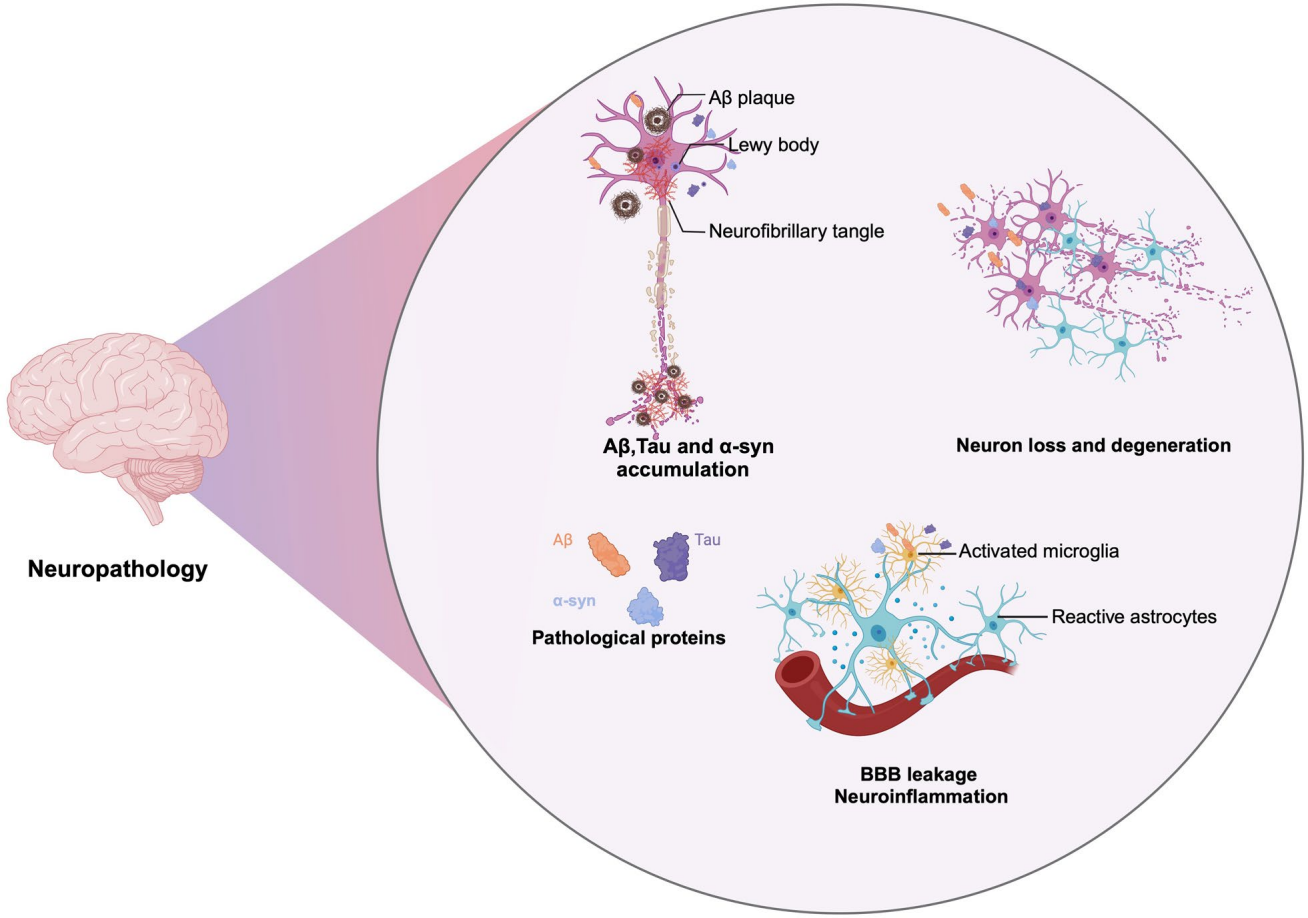
The pathogenesis of neurodegenerative diseases caused by peripheral pathological proteins. After these peripheral pathological proteins enter the brain, they can activate microglia and astrocytes, triggering neuroinflammation, and are involved in the formation of Aβ plaques, neurofibrillary tangles, and Lewy bodies. They also lead to neuronal loss and degeneration. Additionally, disruption of the BBB by these proteins creates a vicious cycle, exacerbating neuropathology

Once transported to the CNS, peripherally derived Aβ and Tau may impair brain function through several pathways. First, Aβ exacerbates the formation and deposition of Aβ fibrils in the brain, which may be one of the main sources of Aβ plaques [13, 130]. Similarly, peripheral Tau can cross into the CNS and contribute to the spread of neurofibrillary tangles [127, 131]. Second, the aggregates of peripheral Aβ and Tau exert certain neurotoxicity and can directly induce neuronal death. Furthermore, gut-derived Aβ and Tau can trigger neuroinflammation, activating microglia, thereby promoting neuronal damage and affecting functions of the CNS [130, 141]. Finally, neuroinflammation induced by intra-gastrointestinal Aβ and Tau can further damage the BBB with a vicious cycle, allowing more peripheral Aβ to enter the brain tissue [120]. In summary, peripherally derived Aβ may negatively affect the CNS through multiple pathways, leading to the progression of AD neuropathology (Fig. 3).

### PD

The gastrointestinal tract not only produces large amounts of Aβ and Tau but also generates α-syn, the pathological protein of PD [142, 143]. An increasing body of research has highlighted the importance of bidirectional communication along the gut-brain axis in the progression of PD [143]. Misfolded α-syn produced in the gut can propagate to the brain via the vagus nerve, where it can seed the formation of new Lewy bodies and contribute to the progression of the disease [144]. Furthermore, peripheral inflammation and gut dysbiosis may promote the misfolding and aggregation of α-syn, exacerbating the disease process [145].

The newly formed Lewy bodies lead to the degeneration of dopaminergic neurons in the substantia nigra, resulting in motor symptoms such as tremor, rigidity, and bradykinesia [146]. Instead of gut-derived α-syn, Yang et al. revealed that erythrocyte-derived α-syn can enter microglia and activate them, triggering neuroinflammation [139]. Additionally, erythrocytic α-syn can accumulate in astrocytic end-feet, regulating glutamate homeostasis and the release of vascular endothelial growth factor A (VEGFA) and inducible nitric oxide synthase (iNOS). Elevated levels of VEGFA and iNOS can disrupt the tight junctions between microvascular endothelial cells, compromising the BBB integrity [140, 147]. These effects collectively contribute to the occurrence and progression of neurodegenerative diseases such as PD **(**Fig. 3**)**.

### Other neurodegenerative diseases

The roles of peripheral Aβ, Tau, α-syn, and TDP-43 in other neurodegenerative diseases, such as ALS, FTD, Lewy body dementia, and MSA, are poorly understood. Furthermore, although TDP-43 is widely expressed in peripheral tissues and shows abnormal expression in the muscles of ALS patients [148], no studies have provided evidence that TDP-43 can enter the brain. Given the similarities in protein misfolding and aggregation across these disorders, it is plausible that peripheral sources of these proteins may also contribute to other neurodegenerative pathogenesis [149]. However, further research is needed to confirm this.

## Peripheral immune dysfunction induced by peripheral proteinopathies in neurodegenerative diseases

In addition to causing neuropathology in the brain, recent studies have found that these peripheral pathological proteins can also induce dysfunction of the peripheral immune system, thereby exacerbating the progression of neurodegenerative diseases. Recent studies have shown that peripheral accumulation of pathological proteins can activate the peripheral immune system, causing chronic inflammation and accelerating the progression of neurodegenerative diseases [150]. Research has found presence of a large amount of Aβ aggregates and reduced levels of monocytes in the plasma even before the prodromal stage of AD [151], which may be related to an increased number and activity of monocyte-derived osteoclasts in the blood [152]. Though still controversial (see early discussion), peripheral Aβ has been shown to contribute to the pathogenesis of AD by activating innate immune cells, which promote the secretion of pro-inflammatory cytokines and molecules that increase BBB permeability [153]. Single-cell RNA-seq revealed that the abundance of human peripheral immune cells is regulated by Tau-related pathophysiological changes [154]. A study involving 1107 participants indicates that Aβ and Tau may be associated with changes in neutrophils and lymphocytes in the periphery and the neutrophil/lymphocyte ratio in AD [155], although the mechanism remains to be investigated. Our research team demonstrated that oligomeric α-syn derived from red blood cells can enter monocytes via receptor-mediated endocytosis, activating LRRK2 and leading to immune hyperactivation of monocytes in the circulatory system [156]. TDP-43 is also expressed in immune cells in peripheral blood, particularly in monocytes [157]. EVs containing TDP-43 induce activation of peripheral monocytes and functional deregulation of monocytes in ALS [158]. These studies underscore the important role of peripheral pathological proteins in neurodegenerative diseases not only by modulating central pathology but also through regulation of peripheral homeostasis, offering new approaches for the diagnosis and treatment of neurodegenerative disease.

## Potential therapeutic targets

Given the potential roles of peripheral pathological proteins in the progression of neurodegenerative diseases, an important therapeutic strategy is to accelerate the clearance of these peripheral pathological proteins. Currently, several groups are attempting various approaches to reduce peripheral Aβ in order to improve AD pathology (Table 2). Monoclonal antibodies including Lecanemab and Donanemab have been verified to bind Aβ and enhance its clearance from the brain through several mechanisms, such as facilitating microglial phagocytosis, dissolving fibrillar Aβ, and activating the complement system [159–162]. However, these antibodies also bind to peripheral Aβ, facilitating Aβ efflux from CNS via a peripheral sink mechanism, without the need to cross the BBB to clear brain Aβ [163]. It is conceivable that after these antibodies neutralize Aβ in the blood, they may tilt the equilibrium of Aβ between the central and peripheral compartments, assisting the transport of Aβ from the brain to the peripheral blood via the LRP1 transporter [164, 165]. Sun et al. found that clearing Aβ from peripheral blood significantly reduces Aβ deposition in the brains of AD mice [166]. Rejuvenation of the peripheral immune system has also been confirmed to improve the pathology of AD [167]. It should be emphasized that approximately 60% of Aβ in the brain is cleared through its transport to peripheral tissues [33]. The liver and kidneys are the main organs that metabolize and clear Aβ and α-syn from blood. Several studies reported that accelerated clearance of peripheral pathological proteins such as Aβ inhibits disease progression. Conversely, insufficient metabolization in the liver and kidneys can exacerbate the pathology of AD [168, 169]. Moreover, the spleen is also involved in the physiological clearance of circulating Aβ in the periphery [170]. According to this clearance strategy, many techniques, including peritoneal dialysis and hemodialysis, have been explored for their efficacy in decreasing Aβ levels [171–173]. Additionally, some studies have discovered that the liver, as a major organ for clearing peripheral α-syn, could potentially improve the progression of PD pathology by enhancing the liver’s capacity to clear α-syn [92, 174].

**Table 2:** Summary of recent studies on clearing peripheral Aβ in AD

Treatments		Subject	Outcomes	References
Medication	Monoclonal antibodies: Lecanemab; Donanemab	AD patients	Reduce blood and brain Aβ; Recover CSF biomarkers; Slow clinical progression	[159] [160] [161] [162]
	Furosemide	APP/PS1 mice	Reduces blood and brain Aβ; Attenuates AD pathologies; Improves cognitive deficits	[169]
Operation	Hemodialysis	Patient with end-stage renal failure	Reduces brain Aβ accumulation	[171]
	Peritoneal dialysis	Patients with newly diagnosed CKD; APP/PS1 mice	Reduces plasma and brain Aβ; Reduces Tau hyperphosphorylation; Suppresses neuroinflammation; Reduces neuronal and synaptic loss; Rescues behavioral deficits	[172]
	Blood exchange	Tg2576 mice	Reduces cerebral amyloid plaques; Improves spatial memory performance	[173]
	Bone marrow transplantation	APP/PS1 mice	Reduces blood and brain Aβ; Rejuvenates peripheral immunity; Attenuates neuronal degeneration; Inhibits neuroinflammation; Improves behavioral deficits	[166] [167]
	LRP-1 overexpression in liver	APP/PS1 mice	Attenuates cerebral Aβ deposition; Attenuates cognitive impairments	[168]
	Splenectomy	APP/PS1 mice	Increases brain Aβ burden; Aggravates behavior deficits; Aggravates AD pathologies	[170]
	Unilateral nephrectomy	APP/PS1 mice	Increases brain Aβ deposition; Aggravates Tau hyperphosphorylation; Increases neuroinflammation; Increases neuronal loss; Aggravates cognitive deficits	[169]

To effectively target peripheral proteinopathies, we must fully understand their pathological characteristics, transmission routes, and disease-causing mechanisms. This knowledge is essential for developing interventions that specifically target pathogenic forms while preserving the physiological functions of these proteins.

Another crucial strategy is to improve clearance of pathogenic proteins. Enhancing the functions of peripheral organs like the liver and kidneys, improving blood purification methods, and targeting lymphatic pathways are promising approaches to reducing brain proteinopathies. By mitigating the pathological effects of Aβ, Tau, and α-syn, these strategies could potentially slow disease progression.

## Conclusions and perspectives

This review highlights the growing evidence for the peripheral origins of Aβ, Tau, α-syn, and TDP-43 and their entry into the CNS through various routes, including the bloodstream and neural pathways like the vagus nerve. Once in the brain, these proteins contribute to neuronal loss, synaptic inhibition, neuroinflammation, and BBB disruption, leading to the onset and progression of neurodegenerative diseases. Even without entering the brain, these proteinopathies can induce neuropathology by modulating the peripheral immune system. These findings challenge the traditional view of neurodegenerative diseases as solely CNS disorders and underscore the importance of considering the role of peripheral tissues and organs in the pathogenesis. Our review presents a new perspective on neurodegenerative diseases by considering them from the perspective of peripheral proteinopathies, offering new insights and strategies for the prevention and treatment of these diseases.

While our understanding of the involvement of peripheral pathological proteins in neurodegenerative diseases has advanced, many questions remain unanswered, including: (1) the mechanisms underlying the production and release of these proteins in both normal physiology and disease conditions, (2) the detailed pathways through which peripheral pathological proteins enter the brain, particularly the role of the vagus nerve, (3) the pathogenic mechanisms of these proteins in the brain, including their interactions with other pathological proteins, which is crucial for developing targeted therapies and preventive strategies against neurodegenerative diseases, and (4) the role of other peripheral amyloidogenic peptides, such as islet amyloid polypeptides (known as amylin) that can also spontaneously aggregate and cross the BBB, propagating into the brain and contributing to neuropathology [175–177].

Furthermore, efforts should focus on developing effective therapeutic strategies targeting peripheral pathological proteins, including drugs and interventions that specifically target these proteins, as well as strategies to modulate their production and release from peripheral tissues. Through these endeavors, we can deepen our understanding of the pathogenesis of neurodegenerative diseases and pave the way for novel approaches to the prevention and therapy of these debilitating disorders.

## Data Availability

Not applicable.

## Abbreviations

CNS: Central nervous system
AD: Alzheimer’s disease
PD: Parkinson’s disease
MSA: Multiple system atrophy
ALS: Amyotrophic lateral sclerosis
FTD: Frontotemporal dementia
Aβ: Amyloid-β
α-syn: α-synuclein
TDP-43: TAR DNA-binding protein 43
APP: Amyloid precursor protein
BACE1: β-Secretase
NCT: Nicastrin
BBB: Blood–brain barrier
EVs: Extracellular vesicles
RBC-EVs: Red blood cell-derived EVs
